# A Functional Screen Identifies Specific MicroRNAs Capable of Inhibiting Human Melanoma Cell Viability

**DOI:** 10.1371/journal.pone.0043569

**Published:** 2012-08-22

**Authors:** Jos B. Poell, Rick J. van Haastert, Thijs de Gunst, Iman J. Schultz, Willemijn M. Gommans, Mark Verheul, Francesco Cerisoli, Paula I. van Noort, Gregoire P. Prevost, Roel Q. J. Schaapveld, Edwin Cuppen

**Affiliations:** 1 Hubrecht Institute, University Medical Center Utrecht, Cancer Genomics Center, Utrecht, The Netherlands; 2 InteRNA Technologies BV, Utrecht, The Netherlands; University of Connecticut Health Center, United States of America

## Abstract

Malignant melanoma is an aggressive form of skin cancer with poor prognosis. Despite improvements in awareness and prevention of this disease, its incidence is rapidly increasing. MicroRNAs (miRNAs) are a class of small RNA molecules that regulate cellular processes by repressing messenger RNAs (mRNAs) with partially complementary target sites. Several miRNAs have already been shown to attenuate cancer phenotypes, by limiting proliferation, invasiveness, tumor angiogenesis, and stemness. Here, we employed a genome-scale lentiviral human miRNA expression library to systematically survey which miRNAs are able to decrease A375 melanoma cell viability. We highlight the strongest inhibitors of melanoma cell proliferation, including the miR-15/16, miR-141/200a and miR-96/182 families of miRNAs and miR-203. Ectopic expression of these miRNAs resulted in long-term inhibition of melanoma cell expansion, both *in vitro* and *in vivo*. We show specifically miR-16, miR-497, miR-96 and miR-182 are efficient effectors when introduced as synthetic miRNAs in several melanoma cell lines. Our study provides a comprehensive interrogation of miRNAs that interfere with melanoma cell proliferation and viability, and offers a selection of miRNAs that are especially promising candidates for application in melanoma therapy.

## Introduction

Melanoma is a skin malignancy with one of the fastest increasing incidences of all cancer types [1]. Although it is a relatively uncommon form of skin cancer, it accounts for over 65% of skin cancer-related deaths [2]. This is due to the extremely high mortality rate once the cancer becomes metastatic [3]. Up to 90% of melanomas rely on mitogen-activated protein kinase (MAPK) signaling for their proliferative capacity [4]. Constitutive MAPK signaling is most commonly acquired through activating mutations in BRAF and NRAS [5]. Recent advances have been made in targeted therapy of metastatic melanoma by targeting the MAPK pathway, but already many cases of drug resistance have been reported [6], [7], [8]. Therefore, it seems unlikely that these targeted drugs will dramatically decrease mortality, unless they are supplemented by other drugs.

MicroRNAs (miRNAs) are small RNAs that regulate gene expression [9]. Utilizing miRNAs to target specific pathways has demonstrated their therapeutic potential in diverse pathologies, such as aberrant cholesterol homeostasis [10], colon cancer [11], and cardiovascular disease [12]. Indeed, some miRNAs have been reported to mitigate or promote malignant capabilities of melanoma cells [13], [14], [15], [16]. Notwithstanding these accounts, the full potential of miRNAs to stunt melanoma progression has not been exhausted, as a systematic approach to probe for tumor-suppressive miRNAs in melanoma has yet to be applied. Loss of miRNA expression in cancer cells is commonly investigated for a causative role in tumor etiology, but this approach does not directly address the question which miRNAs are able to avert disease progression. Instead, we decided on an unbiased approach, using a genome-scale lentiviral human miRNA expression library to assess each miRNA for its potential to affect melanoma cell viability. The most potent miRNAs were validated independently using synthetic miRNA mimics, and in additional melanoma cell lines, further expanding the potential therapeutic avenues for miRNA-based approaches against deadly melanoma.

## Results

**Figure 1:**
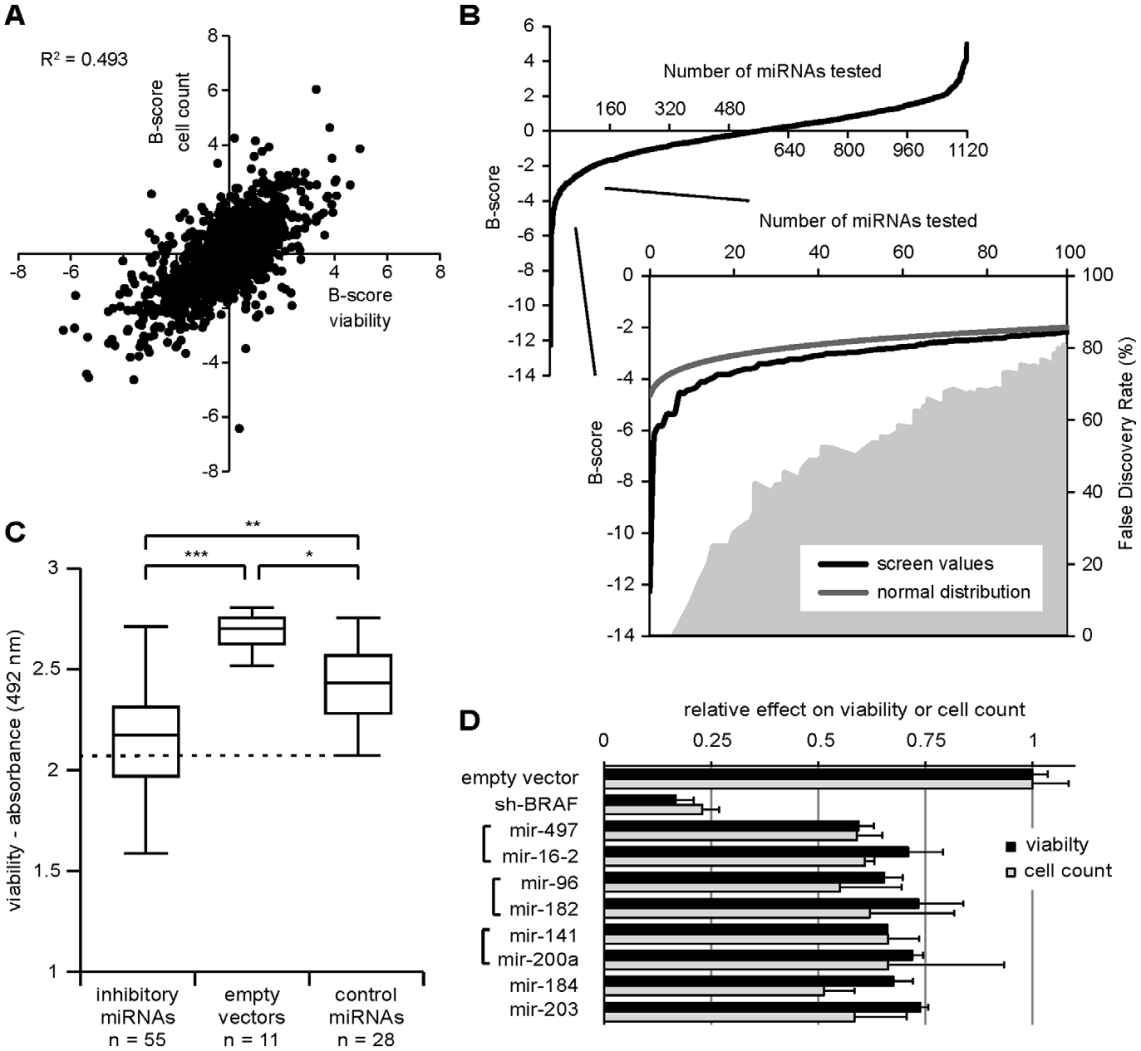
A genome-wide screen for miRNAs that inhibit A375 melanoma cell growth. (A) Inhibition of melanoma growth was measured by means of cell viability and cell count. For each sample a B-score was calculated and B-scores from both assays are plotted against each other. There is a strong correlation between both assays. The B-scores for one miRNA, miR-518b, fell outside the range of the graph: they were −6 for cell count and −12 for cell viability. (B) A comparison with a normal distribution shows that the cell viability screen is sensitive for identifying growth-inhibitory miRNAs. A concomitant estimate of the false discovery rate is shown in grey fill (secondary axis). (C) 55 potential inhibitory miRNAs were tested in a confirmation screen against 11 empty vector samples and a population of 28 miRNAs with small or no effects in the primary screen. Box plots show values between 25^th^ and 75^th^ percentile in boxes, and the outermost values as whiskers. 20 of 55 inhibitory miRNAs scored better than any of the control miRNAs (below dashed line). *p = 6.8*10^−5^, **p = 1.6*10^−6^, ***p = 5.4*10^−10^. (D) Individual hits selected for follow-up, and their relative effect on cell viability. A virus containing a short-hairpin construct targeting BRAF was used as a positive control. Error bars represent standard deviation of three samples.

To systematically identify miRNAs that hinder melanoma cell proliferation, we screened 650 human miRNAs and another 422 candidate human miRNAs for their potential to slow A375 melanoma cell proliferation using a lentiviral miRNA expression library [17]. A375 cells are well-studied malignant melanoma cells that carry the activating BRAF^V600E^ mutation. Two distinct measures of cell growth were assessed in parallel; viability by means of MTS assay [18] and cell number by nuclear staining and automated image analysis. The MTS assay provides a single measure for the viability of all cells in a well, as it depends on the cumulative metabolic activity in the culture, and is therefore strongly correlated with the number of cells in a well [18]. Additionally, it is able to pick up a decrease in cell viability, even when the number of cells is not (yet) affected. For the cell count assay, cells were fixed and stained with Hoechst. Individual cells were identified and quantitated using automated image analysis. The cell count assay therefore gives an end-point measurement of the total accumulated number of cells in a well. In both the MTS assay and the cell count assay, A375 cells were infected in duplicate with a miRNA-containing lentiviral vector and evaluated 6 days after infection. Individual measurements were converted to B-scores ([19], see also Materials and Methods) to standardize measurements from different plates and different assays. B-scores for duplicates in both screens are listed in table S1. The results of the two screens show a strong positive correlation between viability and cell number (figure 1a). Using the MTS readout, more miRNAs scored a B-score of −3 than expected by chance (figure 1b), indicating inhibitory miRNAs could be identified with high confidence. This was not the case when only using cell counts as a measure of inhibition (figure S1). Given the good correlation between the two screens, and the superior performance of the viability measure over cell count alone (figure S1), we selected miRNAs for further testing if they scored an average B-score of −3 in the viability screen. Additional miRNAs were included for follow-up evaluation that scored well (B-score<−2) in the viability screen and the cell count screen (B-score<−3) or scored well in the viability screen (B-score<−2) and had low virus titer (data not shown).

The 55 selected miRNAs were confirmed in a second-round screen using both the MTS assay and the cell count assay. In this experiment the inhibitory miRNAs were compared to a set of 11 individually prepared empty vector samples and a population of 28 control miRNAs considered to have no effect in the initial screen (figure 1c). The 55 selected miRNAs inhibited viability significantly better than the empty vectors and the control miRNAs. Similar results were obtained with the cell count assay (figure S2). 49 out of 55 miRNAs caused lower viability readouts than 95% of the control miRNA population, and 20 inhibitory miRNAs scored better than any of the control miRNAs. The false discovery rate within these 20 miRNAs was 8% (i.e. only 1 false positive). Interestingly, the control miRNAs also decreased cell viability when compared to the empty vectors.

Given the high number of inhibitory miRNAs, we further narrowed our selection of miRNAs for follow-up evaluation. We chose the top scoring miRNA constructs from the second-round, confirmation screen: mir-497, mir-96, mir-141, and mir-184 (figure 1d). We also included three miRNA constructs encoding miRNAs belonging to the same families of the top hits, which were also represented in the 20 best-performing miRNA constructs of the confirmation screen: mir-16-2, mir-182, and mir-200a, which are related to mir-497, mir-96, and mir-141 respectively (figure 1d). Finally, we selected mir-203 from the list of 20 high-confidence hits, because it is known to play a crucial role in skin differentiation [20]. Since A375 cells are oncogene-addicted to mutated BRAF [21], an shRNA targeting BRAF (shBRAF) was used as positive control for strong impediment of A375 viability [22]. All selected miRNAs reduced viability by over 25% after lentiviral introduction (figure 1d).

In the previously described lentiviral transduction experiments, miRNAs are ectopically expressed using a constitutive promoter. After transduction with a particular construct, expression of the enclosed miRNA is expected to be elevated. To ensure miRNA overexpression, we measured the endogenous expression of all selected miRNAs in empty vector-transduced A375 cells and miRNA-transduced A375 cells by qPCR. Overexpression of all miRNAs was efficiently achieved by lentiviral transduction (table 1). Increase of miR-16 expression was modest, but we note that it is an endogenously abundant miRNA in A375 cells (table 1). To gain a better view of endogenous miRNA expression in A375 cells, we further characterized miRNA expression by small RNA massively parallel sequencing. Indeed, miR-16 comprises a substantially larger fraction of sequence reads than any of the other miRNAs (table 1), a finding that is in line with the limited overexpression of miR-16.

**Table 1:** Endogenous miRNA expression and expression after lentiviral transduction in A375 cells.

*miRNA*	*qPCR endogenous (2^−^* ^Δ*Ct*^ *)*	*qPCR ectopic (2^−^* ^Δ*Ct*^ *)*	*overexpression (fold)*	*sequence reads per million*
miR-497	2.57*10*^−^* ^7^	1.75*10*^−^* ^5^	68	3.0
miR-16	5.53*10*^−^* ^3^	2.07*10*^−^* ^2^	3.7	1084
miR-96	1.05*10*^−^* ^6^	1.19*10*^−^* ^5^	11	0.48
miR-182	3.01*10*^−^* ^6^	3.01*10*^−^* ^4^	100	30
miR-141	1.66*10*^−^* ^6^	3.94*10*^−^* ^4^	237	0
miR-200a	1.12*10*^−^* ^7^	6.07*10*^−^* ^4^	5.4*10^3^	0.64
miR-184	8.35*10*^−^* ^8^	1.35*10*^−^* ^3^	1.6*10^4^	1.3
miR-203	1.85*10^−8^	1.09*10^−3^	5.9*10^4^	0.16

qPCR data are relative to U6 small RNA. Endogenous: empty vector-transduced, ectopic: miRNA-transduced.

**Figure 2:**
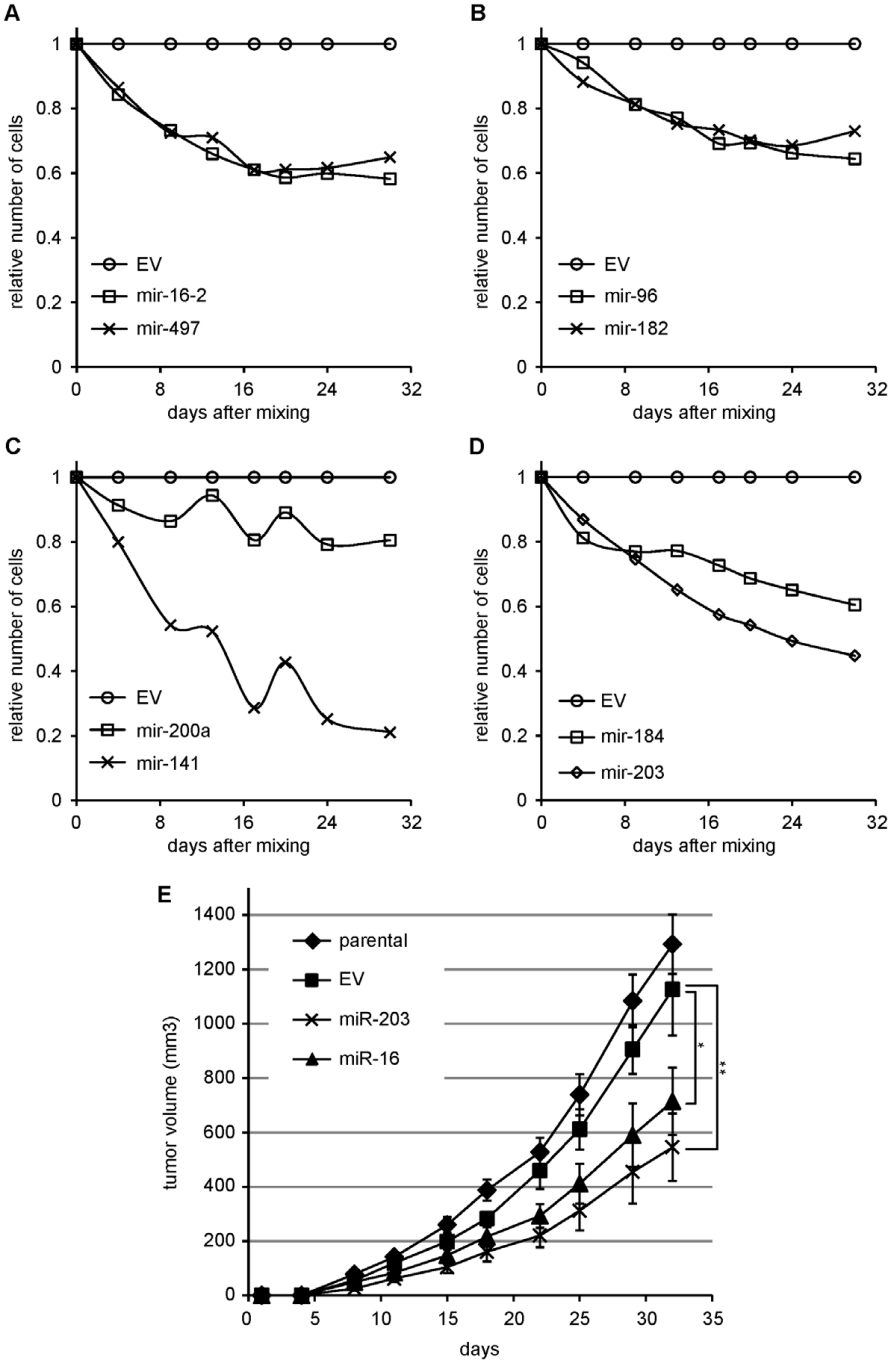
Long-term inhibition of A375 cell growth by miRNAs. (A–D) A375 cells were stably transduced with a miRNA-containing or empty vector (EV) virus and mixed with GFP-expressing A375 cells. Relative number of miRNA-transduced cells was inferred from the ratio of GFP-negative and GFP-positive cells and normalized to relative number of EV-transduced cells. Each panel shows a different subset of transduced miRNAs. A single experiment is shown. (E) Stably transduced cells were injected into nude mice and tumor growth was monitored for 32 days. Error bars represent standard error of the mean, n = 6. *p = 0.078, **p = 0.011.

For effective therapeutic application of miRNAs against cancer, it is important that miRNAs are able to perpetuate their inhibitory effects beyond their initial impact, and potentially to compound their negative influence through indirect effects on downstream processes. To assess more long-term and broader-reaching effects, A375 cells stably expressing an inhibitory miRNA were grown in competition with A375 cells stably expressing GFP, but no ectopic miRNA. The effects of different miRNAs on A375 cell proliferation were inferred from the changes in GFP-positive versus GFP-negative cell ratios. This experimental design allows us to observe cell growth patterns over a prolonged period of time. Since the miRNA-transduced cells grow in the same culture as the GFP-positive cells, many of the potential artifacts associated with changing culture conditions are avoided. Cells stably transduced with inhibitory miRNAs retarded cell growth (figure 2), decreasing cell number 25–80% compared to the empty vector control after 32 days of continuous culture. These results support that introducing an inhibitory miRNA extends beyond a once-only shock to the cells, to achieve a growth-suppressing effect that lasts. We note that for mir-16/497 (figure 2a) and mir-96/182 (figure 2b) the impairment of cell growth diminished over time.

For two miRNAs, miR-16 and miR-203, we examined whether *in vitro* long-term inhibition translated to a reduction in tumor growth in an *in vivo* xenograft model. Parental cells or cells stably expressing an empty vector, a miR-16 construct or a miR-203 construct were injected subcutaneously in nude mice. In line with the results obtained with the *in vitro* culture, both miR-16 and miR-203 reduced tumor growth *in vivo* (figure 2e) when compared to parental A375 cells or empty vector transduced A375 cells.

**Figure 3:**
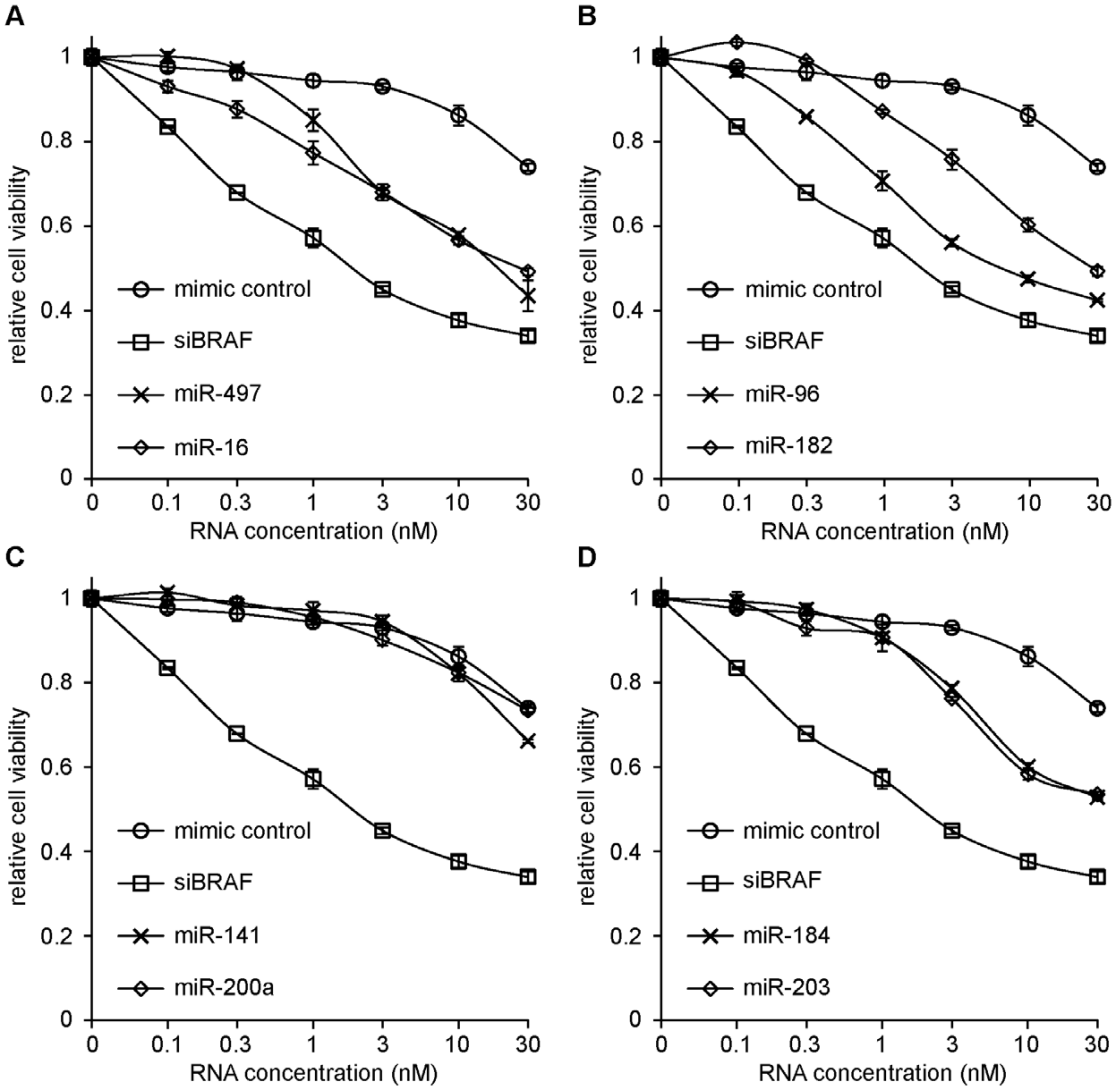
Effect of introduction of synthetic miRNAs on A375 viability. A375 cells were transfected with a range of concentrations of different miRNAs, and 72 hours after transfection viability was measured by means of MTS assay. Effects are compared to a scrambled control and a pool of 4 siRNAs against BRAF (siBRAF) as a positive control for A375 growth inhibition. Specific effects of miRNAs are best observed at concentrations of 10 nM. Each panel shows a different subset of miRNA mimics, although miRNAs were assessed in the same experiment. Error bars represent standard deviation of three samples. A representative of three experiments is shown.

To bring additional evidence that the observed effects are due to the miRNA itself and not extraneous aspects of lentiviral transduction, we tested whether we could reproduce the effects with synthetic miRNA mimics. Additionally, the use of synthetic RNAs allows direct comparison of the potency of different miRNAs at specific concentrations, which is not possible with the lentiviral overexpression system. We examined the effect of introducing synthetic miRNAs at concentrations between 0.1 and 30 nM (figure 3). Viability was measured by MTS 3 days after transfection. Inhibitory miRNAs are compared to a scrambled RNA sequence control, and a pool of 4 siRNAs targeting BRAF (siBRAF) as positive control. Transfecting a scrambled RNA sequence control only affected viability at the highest concentration of 30 nM, while the previously identified inhibitory miRNAs began limiting A375 viability at concentrations around 1 nM. In comparison to the positive control, siBRAF, these miRNAs required higher concentrations to achieve similar effects. The most-specific effects of miRNAs were typically found at 10 nM in A375 cells. It is noteworthy that miR-141, and miR-200a, which had strong effects when introduced by lentivirus, had very little effect when introduced as synthetic mimics.

**Figure 4:**
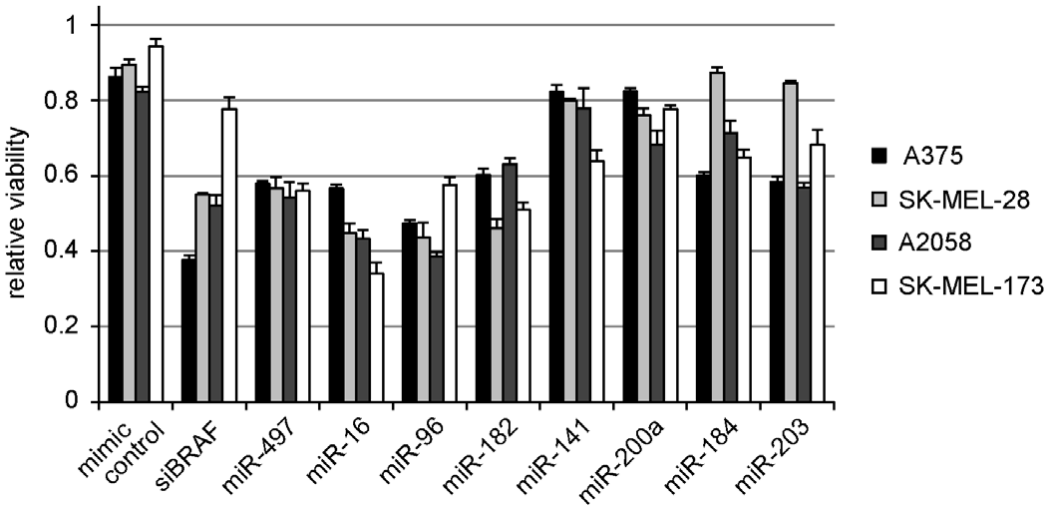
Comparison of miRNA-induced effects in several melanoma cell lines. Cells were transfected with 10 nM (A375 and A2058) or 30 nM (SK-MEL-28 and SK-MEL-173) RNA and cell viability was measured 72 hours after transfection. Data are plotted relative to a mock-infected control. Error bars represent standard deviation of three samples.

To further evaluate the potential utility of the miRNAs identified specifically against melanoma, we tested the inhibitory miRNA mimics in three additional malignant melanoma cell lines, SK-MEL-28, A2058, and SK-MEL-173. We measured miRNA-induced inhibition of viability at a concentration range between 0.1 and 30 nM, and determined that optimal concentrations were 30 nM for SK-MEL-28 and SK-MEL-173 and 10 nM for A2058 (data not shown). A comparison of the inhibitory effects of miRNAs on these cell lines and A375 is given in figure 4. miRNAs yielded very similar effects in all cell lines, with a notable exception for miR-184 and miR-203 in SK-MEL-28. SK-MEL-28 and A2058 proved less sensitive to knockdown of BRAF than A375, even though all three cell lines have the BRAF^V600E^ mutation. As expected, SK-MEL-173 is barely sensitive to knockdown of BRAF, since it carries an activating NRAS mutation, which may provide compensatory proliferation and survival stimulation via the PI3K pathway [23], causing the SK-MEL-173 cells to be less dependent on the MAPK signaling pathway than the other melanoma cell lines. Our data suggest that the miRNAs we have investigated act irrespective of BRAF mutational status, although the number of cell lines tested is insufficient for definitive statements. The data across different melanoma cell lines indicate that the miR-15/16/497 and miR-96/182 family members are the strongest inhibitors of cell viability when introduced as synthetic RNA.

**Figure 5:**
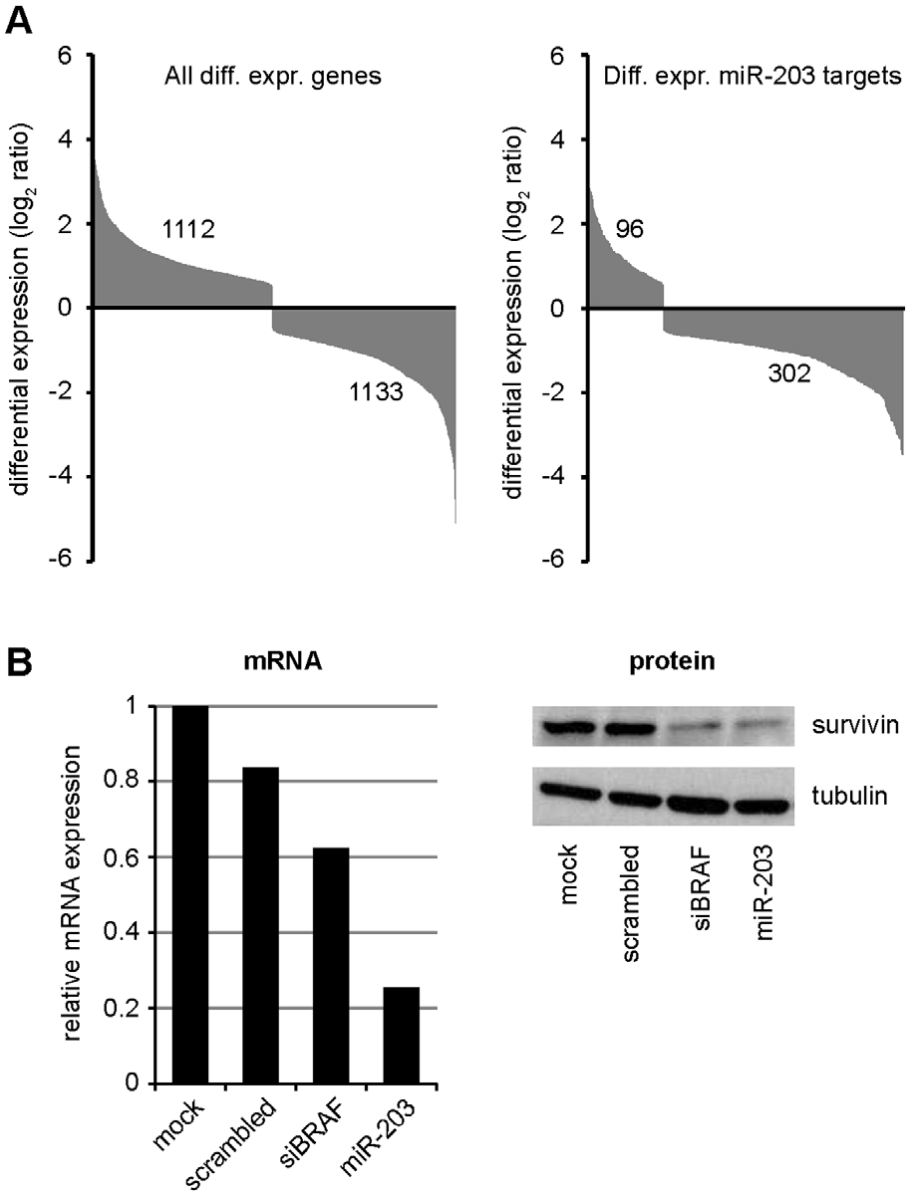
Transcriptome analysis after miR-203 transfection. (A) A375 cells were transfected with either miR-203 or scrambled control and the transcriptome was quantified by RNA-Seq. All differentially expressed genes are plotted in the left graph, while only the differentially expressed genes containing miR-203 target sites are plotted in the right graph. Genes with miR-203 target sites are much more likely to be downregulated after miR-203 overexpression, and downregulated genes are highly enriched for genes with miR-203 target sites (p<0.0001). (B) One of the differentially expressed genes after miR-203 transfection is BIRC5. Repression was examined at both the mRNA and the protein level by qPCR (left) and Western blot (right) respectively. The BIRC5 transcript and its protein product survivin are both reduced after miR-203 transfection, but also after siBRAF transfection.

While the primary focus of this report is to describe the miRNAs best capable of inhibiting melanoma growth, a further understanding of the molecular effects of the individual miRNAs is a crucial next step in assessing a miRNA’s potential in therapeutic applications. Determination of the cellular targets of miRNAs will reveal the mechanism behind the miRNA’s efficacy. Additionally, identification of a miRNA’s “targetome” can be used to anticipate side effects when the miRNA is applied as a therapeutic. We have explored the effects of one of the miRNAs, miR-203, on the transcriptome of A375 cells. After transfection with miR-203, we observed a very strong enrichment of miR-203 targets in the downregulated genes (figure 5a), a phenomenon previously observed for several miRNAs [24]. The significantly downregulated mRNAs with target sites for miR-203 provide an excellent subset of targets to unravel the miRNA-induced effect. As an example, we found BIRC5, the gene encoding for survivin and predicted to be a direct target of miR-203, to be downregulated after miR-203 transfection. Survivin is an antiapoptotic protein important in melanoma biology [25], and its regulation by miR-203 can explain the reduced proliferation seen after miR-203 overexpression. Transfection of miR-203 caused a strong reduction of survivin protein, as observed by Western blot, verifying the measurements on the transcript level (figure 5b). Together, these data provide an initial step to a more complete understanding of the mechanisms by which miR-203 restricts melanoma cell growth, and they exemplify how transcriptome analysis can be employed to unravel functions of miRNAs of interest.

## Discussion

Treatment of melanoma requires new molecules with different mechanism of action able to bypass the current drug resistance. Here, we have identified and assessed the effects of novel miRNAs on cell growth or viability in four separate approaches: short-term effects after lentiviral transduction (6 days), *in vitro* long-term effects after stable lentiviral transduction (32 days), *in vivo* long-term effects after stable lentiviral transduction (35 days) and short-term effects after introduction of a synthetic miRNA (3 days), the latter being applied to additional malignant melanoma cell lines. Although the effects on cell growth were always qualitatively consistent, marked differences were observed between the effects in the different approaches. While miR-141 and miR-200a efficiently slowed cell growth after lentiviral transduction, they had little effect as synthetic RNAs. We cannot exclude that the lentiviral construct produces a transcript that results in a different miRNA in addition to the standard, annotated mature miRNA. miR-141 and miR-200a are not endogenously present in A375 cells (table 1), therefore we could not address this question for those miRNAs with our small RNA sequence data from A375 cells.

The differences between experimental approaches may have important implications for the development of miRNAs as therapeutics. miRNAs we found to be specifically potent in the short-term viability assays were not necessarily the strongest miRNAs to inhibit long-term cell growth. For example, miR-141 and miR-200a were found equally potent in the confirmation screen (figure 1d), but miR-141 performed significantly better than miR-200a in the long-term assay (figure 2c). We expect that some miRNAs affect pathways for which the cell can compensate, blunting the efficacy of the miRNA in the long term. Other miRNAs may target non-redundant genes that are rate-limiting for cell growth. Such miRNAs can achieve sustained reduction of melanoma growth. Measurements of cell numbers over a longer period of time are crucial to distinguish between brief and lasting consequences of miRNA overexpression. A long-lasting effect on cell growth represents a particularly beneficial trait for a miRNA that is considered for cancer therapy. We have examined long-term effects of lentivirally transduced miRNAs expressed from their genomic backbone, and found differential efficacy of miRNAs over time. We have as yet fewer evidence for long-term potency of synthetic miRNAs; this requires additional in vitro and in vivo experimentation.

In our survey of melanoma-hindering miRNAs, as expected, we found miRNAs with precedence. One of these miRNAs, miR-203, was originally described as a regulator of skin differentiation [20], and has more recently been shown to inhibit melanoma growth by inducing senescence [26]. Also of note is miR-182, which is reported to be a strong repressor of MITF [27], the master regulator of melanocyte differentiation. This is of particular interest, because MITF is commonly deregulated and is even designated a common oncogene in melanoma [28]. Furthermore, MITF may have a major role in the propensity of melanoma to become metastatic [29]. Paradoxically, ectopic expression of miR-182 has been shown to enhance the invasive capacity of melanoma cells and increase metastases in vivo [16], and suppression of miR-182 decreases the potential of A375 cells to metastasize to the liver [30]. These findings are in line with a recent report describing low MITF levels in melanoma-initiating cells with an increased potential for tumor formation [31]. Since high levels of MITF are associated with differentiation and complete loss of MITF causes apoptosis, both up- or downregulation of MITF can have desirable effects for melanoma treatment, as long as the change in MITF levels is drastic enough [32]. This may prove impossible, since metastatic melanomas are heterogeneous tumors with both a highly proliferative population, characterized by high MITF, and a highly invasive population, characterized by low MITF [33], [34]. We propose when metastasis has already occurred, preferentially inhibiting cell growth outweighs the benefit of fighting the invasive potential of melanoma, and as such, inhibiting MITF may improve patient survival.

Two members from the miR-15/16 family, i.e. miR-16 and miR-497, were also identified in our screen. This highly conserved family of miRNAs is well-known for its tumor suppressive qualities [35]. The miR-15a/16-1 locus on chromosome 13 is deleted in more than half of B cell chronic lymphocytic leukemias [36]. Several cell cycle-stimulating genes have strong validated or predicted targets for the miR-15/16 family, such as Cyclin E [37], Cyclin D1-3 [37], [38], AKT3, and BCL2 [39] and BCL2L. BCL2 has an anti-apoptotic function and acts in synergy with MITF [40]. Therefore, simultaneous inhibition of MITF and BCL2 may be especially potent by sensitizing melanoma cells to apoptosis. Similarly, targeting AKT3 may also prove useful in this respect. AKT3 activity is commonly increased in melanoma [41], and it is directly responsible for resistance to apoptosis [42]. This makes the miR-15/16 family an excellent candidate for anti-melanoma therapy, especially in combination with MAPK pathway inhibitors [42].

The miR-15/16/497 and miR-96/182 families target distinct subsets of genes, both affecting melanoma cell proliferation. Indeed, when used in combination miR-16 and miR-96 (5 nM each) yielded a better outcome than either miRNA transfected separately at 10 nM in both A375 and SK-MEL-28 cells (figure S3). An additive effect was expected, since both miRNAs have the same effect on cell growth, but target different genes. A combination treatment will thus compromise additional pathways. Alternatively, genes with target sites for both miRNA families may be repressed synergistically [43]. Even though combining two or more miRNAs increases the risk of undesirable side effects, it may decrease intensity of the side effects, as each individual miRNA can be used at lower concentration. Though preliminary, our results support investigating and developing multi-miRNA based therapies. In pursuit of this, genome-scale screens to find all potentially beneficial miRNAs are paramount.

We identified a set of miRNAs that are particularly effective in inhibiting melanoma expansion. These miRNAs do not have an abruptly toxic effect on the melanoma cells, but may assist in the attenuation of cancer growth and sensitize cells to other therapeutics. Conclusive evidence for this will have to come from additional in vivo experiments and combination therapy approaches. miRNAs do not have a singular effect on cells, but potentially repress hundreds of genes to varying degrees [44], affecting several cellular pathways. If a tumor-suppressive phenotype of a miRNA is caused by targeting a large set of genes, this may create a major obstacle for the development of resistance. Thus, miRNAs may prove valuable components of combination therapies for metastatic melanoma. This therapeutic miRNA identification strategy may be extended to other cancers.

## Materials and Methods

### Lentiviral Constructs

Construction and validation of the lentiviral library have been presented in detail elsewhere [17]. In brief, human miRNA sequences were cloned into a lentiviral expression construct (pCDH-CMV-MCS-EF1-Puro, System Biosciences) from their genomic background, including ∼100 bp flanking the precursor hairpin. An expression construct with EGFP was cloned by excision and ligation of the EGFP sequence from pEGFP-N1 (Clontech). An shBRAF construct was made by hybridizing the following oligonucleotides [22]:

shBRAF-fwd gatccagaattggatctggatcatttcttcctgtcagaaaatgatccagatccaattcatttttg

shBRAF-rev aattcaaaaatgaattggatctggatcattttctgacaggaagaaatgatccagatccaattctgy

The hybridized oligonucleotides were ligated into pSIH-H1-Puro (System Biosciences). Production of lentiviral particles was executed at System Biosciences. Lentiviral particles were provided in separate tubes at concentrations generally between 1*10^8^ and 5*10^9^ infectious units per mL (IFU/mL).

### Cell Culture and Viral Infections

A375 cells were acquired from the Hubrecht Institute in-house cell line repository and SK-MEL-28 cells were purchased from the ATCC. Cells were propagated at 37°C and 5% CO_2_ in DMEM-glutamax (GIBCO) supplemented with 10% fetal bovine serum (FBS, Sigma) and 1% Penicillin-Streptomycin (Invitrogen). For short-term assays with lentiviral infections, 1000 cells were seeded in 100 µL 5% FBS medium per well of a 96-well plate. Edge wells (wells in rows A and B and columns 1 and 12) were excluded from experimentation to avoid edge effects of incubation. Six hours after seeding, cells were infected with a mix of 0.5 µL virus supernatant, 0.6 µL 1 mg/mL polybrene (Sigma) and 8.9 µL PBS0 (GIBCO). 24 hours after infection, medium was replaced with 150 µL fresh 5% FBS medium. Six days after infection, samples were subjected to an MTS assay or fixed for cell counting (see below). For the competition experiment, infections were scaled up to 6-well plates and samples were infected with 5 µL virus supernatant. 24 hours after infection, medium was replaced with 5% FBS medium containing 1 µg/mL puromycin. Cells were puromycin-selected for 3 days, after which GFP-positive and GFP-negative cells were mixed approximately 1∶1. Cell culture was continued on 1 µg/mL puromycin for the duration of the experiment. When cells were passaged, the surplus of cells was analyzed by flow cytometry (FACSCalibur, BD Biosciences) to determine the ratio of GFP-positive and GFP-negative cells.

### RNA Transfections

Cells were propagated and seeded as described above, except 2000 cells were seeded instead of 1000 cells. 16 hours after seeding, cells were transfected with 20 uL Opti-MEM (Invitrogen) containing 3 uL X-tremeGENE (Roche) (unless indicated otherwise), and the indicated amount of Pre-miR miRNA precursor molecule (Ambion), ON-TARGET*plus* SMARTpool BRAF (Dharmacon), or Pre-miR negative control #2 (Ambion). Cells were subjected to an MTS assay three days after transfection.

### MTS Assay

At the indicated time points, medium was replaced with 100 µL fresh 5% FBS medium and 30 µL MTS One Solution (Promega) per well. Absorbance at 492 nm was measured 4 hours after addition of MTS.

### Cell Count Assay

At the indicated time points, 100 µL PBS0 with 8% PFA was added to each well. Cells were fixed for 15 minutes at room temperature and subsequently washed with 100 µL PBS0. Cells were stained for 10 minutes in 100 µL PBS0 containing 0.5 µg/mL Hoechst 33342 (Sigma). Cells were washed twice with PBS0 and kept at 4°C. A relative cell count per well was measured on a Cellomics ArrayScan VTI using the accompanying software by counting nuclei in 4 fields per well under 10x magnification. Nuclei were identified by the software as shapes with a contiguous Hoechst stain.

### Statistical Analysis

In the primary miRNA screen, values of each plate were assessed for intraplate biases per row and per column. No intraplate biases were observed, so corrections were deemed unnecessary (note that edge wells were excluded from experimentation). For each sample a B-score was calculated as follows. First, for each value the absolute deviation from the median of the plate was listed. From this list, the median value constitutes the median absolute deviation or MAD of the plate. The B-score of a sample with value X was then calculated as (X-median)/MAD. B-scores were calculated for all samples in the cell viability screen and the cell count screen. False discovery rates were calculated by dividing the number of hits expected by chance with the observed number of hits. Expected number of hits was calculated as the probability of the cumulative fraction multiplied with the number of tested samples.

### miRNA qPCR

10,000 A375 cells were seeded in 500 µL of a 24-wells plate. Viral transduction was done as described. 6 days after infection cells were washed with cold PBS and put on ice. 200 uL TRIzol (Invitrogen) was added to each well and total RNA was isolated following the manufacturer’s instructions. RNA concentration was quantitated by Qubit RNA assay kit (Invitrogen) according to the manual. miRNA qPCR was performed as described by Chen et al. [45]. Each miRNA assay requires a miRNA-specific stem-loop (SL) primer for reverse transcription, and a miRNA-specific forward primer and universal reverse primer for PCR. U6 requires an additional specific reverse primer. Primers used for qPCR:

SL-U6: 5′-GTCATCCTTGCGCAGG-3′

U6 Forward: 5′-CGCTTCGGCAGCACATATAC-3′

U6 Reverse: 5′-AGGGGCCATGCTAATCTTCT-3′

Universal reverse primer: 5′-GTGCAGGGTCCGAGGT-3′

SL-miR-497: 5′-GTCGTATCCAGTGCAGGGTCCGAGGTATTCGCACTGGATACGACACAAAC-3′

Forward-miR-497: 5′-TGCCAGCAGCAGCACACTGTGGT-3′

SL-miR-16: 5′-GTCGTATCCAGTGCAGGGTCCGAGGTATTCGCACTGGATACGACGCCAA-3′

Forward-miR-16: 5′-GCCCGCTTAGCAGCACGTAAATATT-3′

SL-miR-96: 5′-GTCGTATCCAGTGCAGGGTCCGAGGTATTCGCACTGGATACGAAGCAAA-3′

Forward-miR-96: 5′-GCCCGCTTTTGGCACTAGCACATTTT-3′

SL-miR-141: 5′-GTCGTATCCAGTGCAGGGTCCGAGGTATTCGCACTGGATACGACccatct-3′

Forward-miR-141: 5′-TGCCAGTAACACTGTCTGGTAAAG-3′

SL-miR-200a: 5′-GTCGTATCCAGTGCAGGGTCCGAGGTATTCGCACTGGATACGAACATCG-3′

Forward-miR-200a: 5′-GCCCGCTTAACACTGTCTGGTAACG-3′

SL-miR-184: 5′-GTCGTATCCAGTGCAGGGTCCGAGGTATTCGCACTGGATACGAACCCTT-3′

Forward-miR-184: 5′-GCCCGCTTGGACGGAGAACTGATAA-3′

SL-miR-203: 5′-GTCGTATCCAGTGCAGGGTCCGAGGTATTCGCACTGGATACGACTAGTG-3′

Forward-miR-203: 5′-GCCCGCTGTGAAATGTTTAGGACCA-3′

### Western Blot and qPCR of BIRC5

A375 cells (1.5×10^5^) were seeded in a 6-wells plate in DMEM+10%FCS. The next day cells were transfected with miRNA mimic control #2 (Ambion), BRAF siRNA pool (Dharmacon) or miR-203 mimic (Ambion), at 100 nM concentration using RNAiMAX (Invitrogen). The following day, medium was replaced with fresh DMEM+10%FCS. Seventy-two hours after transfection, medium was aspirated and cells were washed once with PBS. Cells were lysed in RIPA buffer (50 mM Tris pH 8, 150 mM NaCl, 1% NP-40, 0.5% Sodium deoxycholate, 0.1% SDS) supplemented with Protease Inhibitor Cocktail (Sigma), according to the manufacturer’s instructions. 15 µg of protein was loaded on a 7.5% polyacrylamide gel (Bio-RAD) and transferred to PVDF (Millipore). Membranes were incubated with antibodies for β-tubulin and BIRC5 (Santa Cruz). 25 µL of lysate was used for RNA extraction in 1 mL TRI-Reagent (Sigma). 1 µg of RNA was reverse transcribed, and 5% of the resulting reaction product was used in a single qPCR reaction with either BIRC5 or GAPDH Taqman qPCR assays. Both assays were performed in triplicate. Expression of BIRC5 was normalized to GAPDH expression.

### *In vivo* Study with Stably Transduced Cell Lines

Of each cell line, including the parental, 3×10^6^ cells were injected subcutaneously into female immunodeficient NMRI-nu/nu nude mice (n = 6 per group) and tumor growth followed for 32 days (tumor size measured twice weekly). At necropsy, primary tumors were removed, and weight and volume determined.

### Small RNA Massively Parallel Sequencing

The deep-sequencing library was prepared as described previously [46]. In brief, the small RNA fraction between 18–28 nt was isolated from total RNA of A375 cells. A synthetic adaptor was ligated on both sites of the small RNA molecules, followed by first strand cDNA synthesis. The cDNA was subsequently PCR-amplified with adaptor-specific primers. The generated deep-sequencing library was analyzed by massively parallel sequencing on the Solexa system (Illumina) and the reads were submitted to the miRIntess small RNA analysis pipeline [47] (InteRNA Genomics BV, www.interna-genomics.com). Numbers in table 1 represent sequence reads mapping to the miRNA per million mapped reads. 56.6% of all mapped reads in A375 sequencing data mapped to a known miRNA sequence.

### RNA-Seq on Transfected A375 Cells

RNA-Seq libraries were created for Solid sequencing. A375 cells were plated in six wells at 1.5×10^5^ cells/well in 6-wells plates. After overnight attachment the cells were transfected overnight with miR-203 mimics (Ambion) or Pre-miR™ miRNA Precursor Molecules - Negative Control #2 (ambion AM17111) both at a concentration of 100 nM and 12 ul X-tremeGENE siRNA Transfection Reagent per well (Roche). After 72 hours, RNA was isolated using TRIzol® reagent according to manufacturer’s protocol. Purified Total RNA concentration was measured using The Qubit® Fluorometer and 30 µg total RNA was used to create RNA-Seq libraries. Isolation of mRNA was performed using micro polyA purist kit (Ambion AM1919) and mRNA only kit (Epicenter MOE51024). Samples were prepared for sequencing using the SOLiD™ Total RNA-Seq Kit (applied biosystems 4445374). Relative expression was calculated as the ratio of relative reads mapping to a gene in the miR-203-transfected sample and the relative reads mapping to a gene in the control-transfected sample.

## Supporting Information

Figure S1 **Comparison between cell viability and cell count assay.** Results of the cell viability screen by MTS assay are depicted on the left, and results of the cell count screen by Hoechst assay (nuclear count) are depicted on the right. For all individual measurements a B-score was calculated (see materials and methods). Top panel: correlation of duplicate B-scores is shown. The middle panel displays the range of B-scores of all miRNAs tested. The bottom panel zooms in on 100 miRNAs with the lowest B-scores. The graph compares the distribution of B-scores with a normal distribution converted to B-scores (black and grey lines respectively, primary axis), which is used to calculate the false discovery rate (grey fill, right axis).
(TIFF)

Figure S2 **Confirmation screen: cell count assay.** As in figure 1c, but results from cell count assay instead of cell viability assay. *p = 8.8*10^−3^, **p = 3.3*10^−5^, ***p = 7.6*10^−6^.
(TIFF)

Figure S3 **Combinations of miRNAs can cooperate to decrease cell viability.** A375 cells and SK-MEL-28 cells were transfected with a combination of miR-16 and miR-96 (5 nM each) or a miR-16 and miR-96 separately (5 or 10 nM). The combination always scored better than the individual mimics, decreasing cell viability an additional 5–10%.
(TIFF)

Table S1 **B-scores of all miRNAs in the cell viability and cell count screen.**
(XLS)
