# Correction: A Functional Screen Identifies Specific MicroRNAs Capable of Inhibiting Human Melanoma Cell Viability

**DOI:** 10.1371/annotation/ebea4bd5-2b96-4842-b110-2f7c156e5060

**Published:** 2013-01-22

**Authors:** Jos B. Poell, Rick J. van Haastert, Thijs de Gunst, Iman J. Schultz, Willemijn M. Gommans, Mark Verheul, Francesco Cerisoli, Andre A.F.L. van Puijenbroek, Paula I. van Noort, Gregoire P. Prevost, Roel Q. J. Schaapveld, Edwin Cuppen

Dr. Andre A.F.L. van Puijenbroek was not included in the author byline. He should be listed as the eighth author and affiliated with InteRNA Technologies BV, Utrecht, The Netherlands. The contributions of this author are as follows: Conceived and designed the experiments, analyzed the data.

