# Supplementary figures and images for: A Functional Screen Identifies Specific MicroRNAs Capable of Inhibiting Human Melanoma Cell Viability
Source: PLoS One. 2012 Aug 22;7(8):e43569. doi: 10.1371/journal.pone.0043569 (PMC3425484; doi:10.1371/journal.pone.0043569)

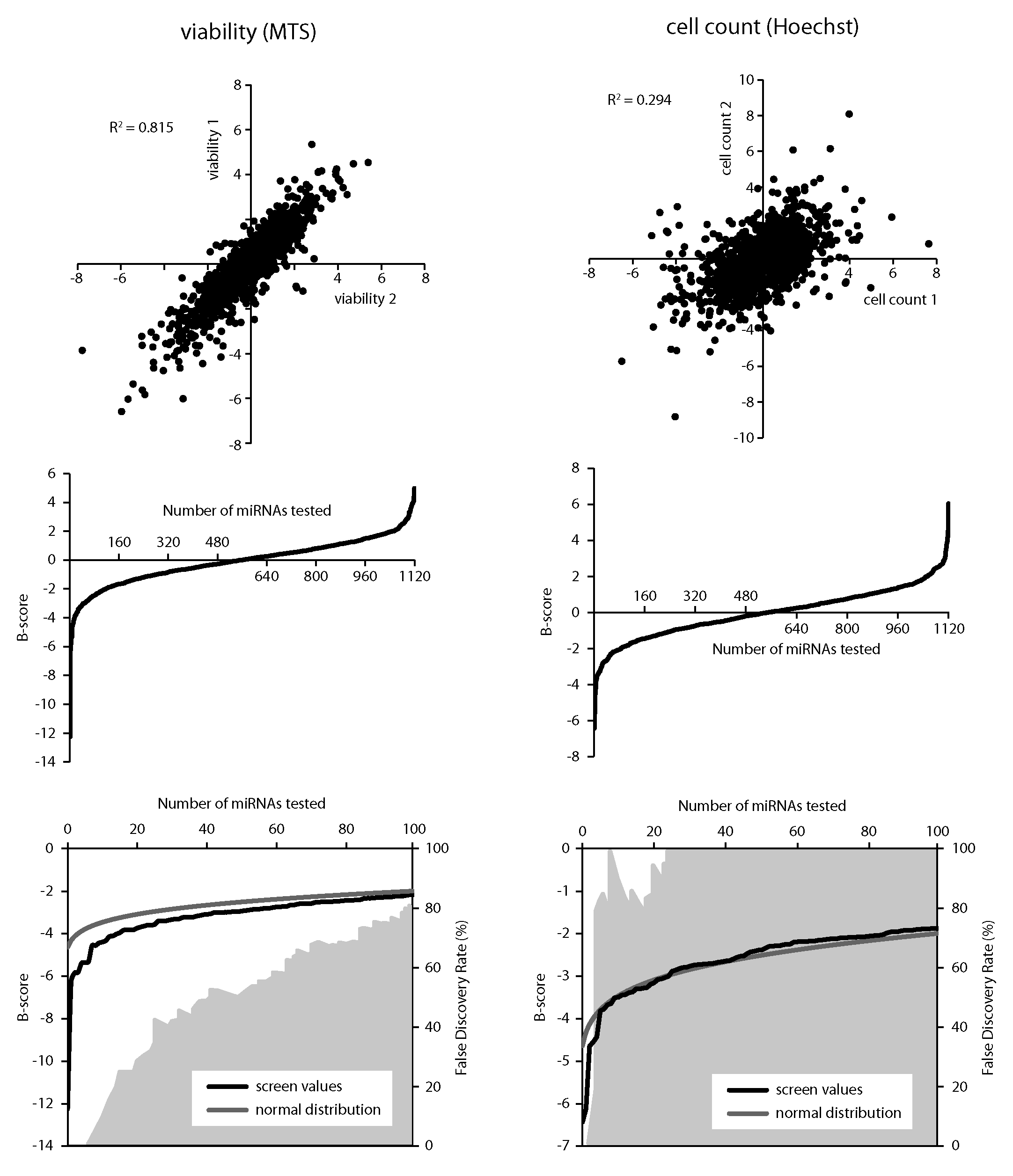

Supplement: Figure S1 — Comparison between cell viability and cell count assay. Results of the cell viability screen by MTS assay are depicted on the left, and results of the cell count screen by Hoechst assay (nuclear count) are depicted on the right. For all individual measurements a B-score was calculated (see materials and methods). Top panel: correlation of duplicate B-scores is shown. The middle panel displays the range of B-scores of all miRNAs tested. The bottom panel zooms in on 100 miRNAs with the lowest B-scores. The graph compares the distribution of B-scores with a normal distribution converted to B-scores (black and grey lines respectively, primary axis), which is used to calculate the false discovery rate (grey fill, right axis). (TIFF) [file pone.0043569.s001.tiff]

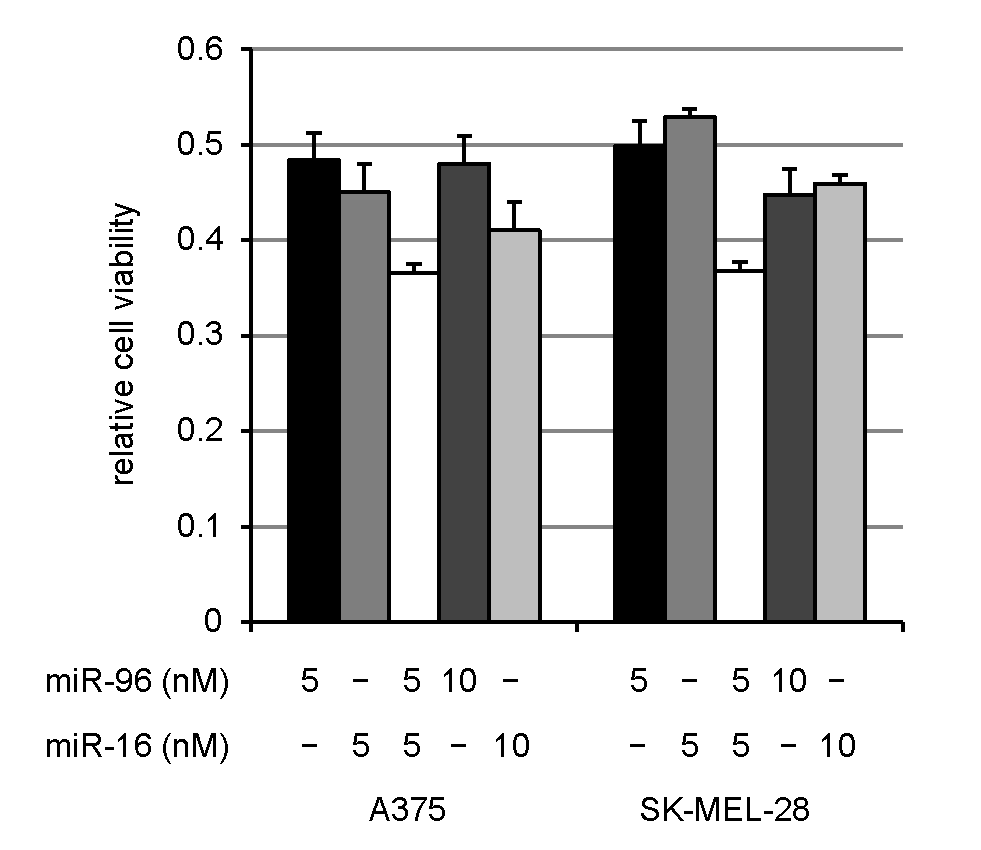

Supplement: Figure S3 — Combinations of miRNAs can cooperate to decrease cell viability. A375 cells and SK-MEL-28 cells were transfected with a combination of miR-16 and miR-96 (5 nM each) or a miR-16 and miR-96 separately (5 or 10 nM). The combination always scored better than the individual mimics, decreasing cell viability an additional 5–10%. (TIFF) [file pone.0043569.s003.tiff]
